# Three-dimensional finite element analysis of translaminar lag screw designs for L5 bilateral spondylolysis

**DOI:** 10.3389/fbioe.2026.1882221

**Published:** 2026-08-13

**Authors:** Feng Li, Xingguo Tan, Tao Zhang, Hua Liu, Songkai Li

**Affiliations:** 1 Department of Spine Surgery, The 940th Hospital of Joint Logistic Support Force of Chinese People’s Liberation Army, Lanzhou, Gansu, China; 2 Department of Orthopedics, Tangdu Hospital, Fourth Military Medical University, Xi’an, Shaanxi, China; 3 Department of Orthopedics, Shexian Hospital, Handan, Hebei, China; 4 First Clinical Medical School, Gansu University of Chinese Medical, Lanzhou, Gansu, China

**Keywords:** biomechanics, finite element analysis, internal fixation, lumbar spondylolysis, surgical therapy

## Abstract

**Purpose:**

This finite element study aimed to quantitatively compare the biomechanical properties of translaminar lag screws with different diameters and structural designs for the surgical repair of L5 bilateral spondylolysis, to provide evidence for individualized screw selection.

**Methods:**

An intact model of L4-S1 segment (Model A) and a L5 bilateral spondylolysis model (Model B) were established using Mimics, Geomagic, and SolidWorks. Additional models were constructed incorporating translaminar lag screws of varying diameters: 4.5 mm (solid, Model C1; cannulated, Model C2), 4.0 mm (solid, Model D1; cannulated, Model D2), and 3.5 mm (solid, Model E1; cannulated, Model E2). Following model validation, a 400 N axial load and a 10 N m moment were applied to simulate five conditions. Analyzed parameters included range of motion (ROM), maximum displacement, and maximum von Mises stress in the disc, pars interarticularis, and fixation screws.

**Results:**

Compared to the intact model (A), the spondylolysis model (B) showed significant instability. All fixation models (C1-E2) effectively restored ROM to intact levels, regardless of screw diameter or structural design. Larger-diameter (4.5 mm) and solid designs demonstrated slightly better control of model displacement. Increasing diameter significantly reduced stress concentrations within the pars interarticularis and fixation screws. For a given diameter, cannulated screws sustained higher stress than solid screws. The 3.5 mm cannulated screw exhibited the highest screw stress, approaching the yield strength of titanium alloy under rotational loading.

**Conclusion:**

All translaminar lag screw designs can effectively restore stability in L5 spondylolysis. While different diameters and structural designs have a minor impact on stability restoration, they significantly affect implant stress. In this specific patient-derived model, the 4.5 mm solid screw demonstrated the most favorable biomechanical profile, with the lowest stress on the implant and effective control of graft displacement. These findings suggest a potential mechanical advantage for 4.5 mm solid screws; however, confirmation through multi-specimen studies or clinical trials is required before broader clinical recommendations can be made.

## 1 Introduction

Spondylolysis refers to a bony defect, possibly a stress fracture of unilateral or bilateral pars interarticularis of the vertebra and most frequently occurs in the lower lumbar spine, predominantly​ at L5, followed by L4 (Debnath, 2021; Berger and Doyle, 2019; Standaert and Herring, 2000). The concept of direct repair for lumbar spondylolysis was first introduced by Kimura in 1968, involving iliac crest bone grafting at the pars defect without fixation (Kimura, 1968). In 1970, Buck described a technique in which a lag screw was placed transversely through the lamina and pars interarticularis, combined with bone grafting; this procedure is termed the translaminar lag screw technique (Buck technique) (Buck, 1970). The Buck technique, as a representative method of direct segmental repair, has demonstrated feasibility through over 5 decades of clinical application (Snyder et al., 2014; Kim et al., 2012; Bonnici et al., 1991; Debnath et al., 2018). Although the Buck screw has a small diameter and short length, its lag mechanism generates compression across the pars defect, thereby enhancing screw purchase (Fan et al., 2010). The advantage of this technique lies in its ability to apply longitudinal compression between the bone graft and the defect site, restoring bony continuity of the pars, effectively reducing stress on the adjacent intervertebral discs, and substantially preserving segmental mobility (Snyder et al., 2014; Sairyo et al., 2006). However, the clinical application of this technique has remained relatively limited in recent years. Major contributing factors include its technically demanding nature, a steep learning curve, and the absence of standardized guidelines for optimal screw designs. The selection of an inappropriate screw size can result in reduced fixation strength. Furthermore, the small surface area of the pars defect limits the space available for bone grafting, as screw placement occupies a portion of this critical zone; this spatial competition may compromise fusion rates (Menga et al., 2014; Giudici et al., 2011).

Currently, the selection of parameters for translaminar lag screws (including diameter and structural design) relies primarily on empirical judgment and lacks robust quantitative evidence (Liu et al., 2020). Traditional biomechanical studies, often relying on cadaveric specimens or two-dimensional radiographic analysis, are limited in dynamically assessing stress distribution at the screw-bone interface under complex loading conditions and evaluating long-term fatigue performance (De et al., 1999). The advent of three-dimensional (3D) finite element (FE) analysis has provided a powerful tool for the precise analysis of mechanical behavior in internal fixation systems and for the optimization of screw parameters (Ranu, 1982; Jones and Wilcox, 2008; Song et al., 2021). This methodology enables the construction of high-fidelity digital models based on individual anatomy, simulates the spinal biomechanical response under physiological loading, and quantitatively evaluates the influence of different screw parameters on fixation stability, stress distribution, and long-term durability (Ye et al., 2022). Furthermore, it can reveal stress transfer mechanisms within the screw-bone construct, predict potential failure risks, and offer a theoretical basis for optimizing screw diameter and structural design.

Recent advancements in minimally invasive spine surgery have renewed interest in the Buck screw, owing to its inherent compatibility with percutaneous placement techniques (Narendran et al., 2023). Furthermore, its integration with spinal endoscopy, navigation systems, and robotic assistance has facilitated greater procedural minimal invasion, precision, and safety (Higashino et al., 2007; Jamshidi et al., 2023; Tian et al., 2020). Consequently, the further evolution of this technique is contingent upon the systematic refinement of screw designs. This study established a refined 3D FE model of the L4-S1 segment and simulated the biomechanical performance of different translaminar lag screw designs in treating single-level lumbar spondylolysis. The biomechanical evaluation encompassed multiple parameters: the maximum displacement of the functional spinal unit and the pars interarticularis, as well as the maximum stress and stress distribution within the pars interarticularis, intervertebral discs, and the internal fixation system. The aim of this study is to provide quantitative biomechanical evidence to guide the individualized selection of Buck screws in clinical practice.

## 2 Materials and methods

### 2.1 3D FE model construction

This study was approved by the Ethics Committee of the 940th Hospital of Joint Logistic Support Force of Chinese People’s Liberation Army (2024KYLL217). Lumbar CT data (DICOM-format) from a 21-year-old patient were imported into Mimics Research 21.0 (Materialise, Belgium) for preliminary 3D reconstruction and exported as an STL file. This file was processed in Geomagic Wrap 21.0 (Geomagic, United States) to generate a nonuniform rational B-spline (NURBS)-based solid model, which was exported in STEP format and then assembled in SolidWorks 2021 (Dassault, France) together with intervertebral discs and ligaments to construct an intact L4-S1 segment model (Model A). Bilateral L5 pars defects (approximately 3 mm) were created in Model A to establish a spondylolysis model (Model B). Finally, by simulating defect repair with translaminar lag screws, additional 3D FE models were constructed corresponding to different screw designs: 4.5 mm (solid, Model C1; cannulated, Model C2), 4.0 mm (solid, Model D1; cannulated, Model D2), and 3.5 mm (solid, Model E1; cannulated, Model E2). The systematic workflow described above is visually summarized in Supplementary Figure S1.

### 2.2 Material properties and contact settings

Material parameters were assigned, and contact conditions were defined in ANSYS Workbench 21.0 (ANSYS, United States) in accordance with published reference data (Ye et al., 2022). The FE models comprised the lumbar vertebrae L4-L5, sacrum S1, two intervertebral discs, four endplates, four facet joints, seven ligament groups (anterior longitudinal, posterior longitudinal, ligamentum flavum, interspinous, supraspinal, capsular, and intertransverse ligaments), and the translaminar lag screws. Cortical bone, cancellous bone, posterior elements, pars interarticularis, annulus fibrosus, nucleus pulposus, articular cartilage, and the cancellous bone graft at the pars defect were modeled as isotropic, linear-elastic materials. The ligamentous structures were modeled as homogeneous, orthotropic, linear elastic spring elements (Jones and Wilcox, 2008), with their stiffness parameters defined through contact interfaces (Ambati et al., 2015; Denozière and Ku, 2006; Mo et al., 2015). Detailed material properties for each structure are summarized in Table 1.

**TABLE 1:** Material properties of finite element models.

Structure	Elastic modulus/MPa	Poisson ratio	Stiffness (N/mm)
Cortical bone	12,000	0.3	​
Cancellous bone	100	0.2	​
Posterior structure	3,500	0.25	​
Cartilaginous endplate	1,000	0.2	​
Annulus fibrosus	4.2	0.45	​
Nucleus pulposus	1	0.499	​
Pars interarticularis (spondylolytic fibrous tissue)	0.2	0.3	​
Cancellous bone graft	100	0.2	​
Translaminar lag screws	120,000	0.3	​
Anterior longitudinal ligament	​	​	8.74
Posterior longitudinal ligament	​	​	5.83
Ligamentum flavum	​	​	15.75
Supraspinous ligament	​	​	15.38
Interspinous ligament	​	​	0.19
Intertransverse ligament	​	​	2.39
Capsular ligament	​	​	10.85

Regarding contact settings, a non-separation condition was applied to the articular cartilage interfaces and the spondylolytic defect regions, while bonded constraints were adopted for all other contact surfaces, with a contact tolerance of 0.05 mm. Mesh generation was performed using second-order tetrahedral elements. A local element size of 1.5 mm was assigned to the articular cartilage and endplates, refined to 1.0 mm for the fixation constructs, and a global size of 2 mm was adopted for the remaining regions. The overall average mesh quality of the model exceeded 0.8.

### 2.3 Convergence analysis

Unstructured meshing was performed using ANSYS Workbench 21.0. To balance computational accuracy and efficiency, a mesh sensitivity analysis was conducted based on the criterion that a relative difference of <5% in peak stress between successive meshes indicates convergence (Ayturk and Puttlitz, 2011). Four L4–S1 models were constructed with global element sizes of 2.5 mm (Mesh4), 2.0 mm (Mesh3), 1.5 mm (Mesh2), and 1.2 mm with local refinement at the screw-bone interface (Mesh1).

### 2.4 Loading and boundary conditions

**FIGURE 1:**
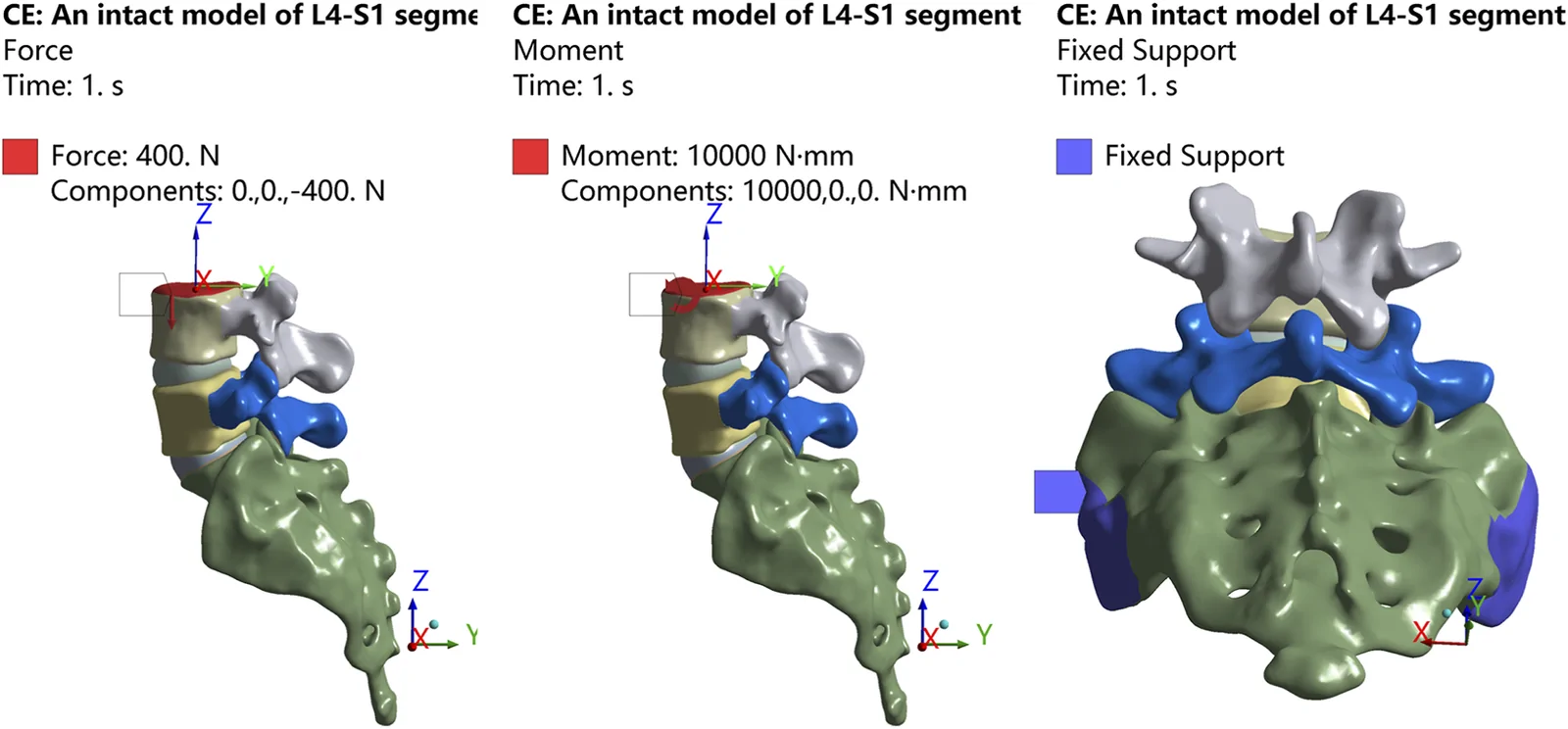
Boundary conditions and loading configuration of the finite element model.

To simulate physiological loading, the sacrum was fully constrained in all degrees of freedom. A 400 N axial compressive force, representing trunk weight, was applied to the superior surface of L4. To assess 3D spinal mobility, a torsional moment of 10 N m was applied to simulate spinal fundamental loading conditions: flexion, extension, rotation, and lateral bending (Figure 1). Although bilateral analysis of the intact model revealed minor asymmetries, the mechanical responses under left- and right-sided loading were fundamentally similar. Since this is a purely biomechanical investigation, assuming structural symmetry is methodologically justified. Consequently, we restricted our detailed analysis to five representative loading conditions: axial compression, flexion, extension, left rotation, and left lateral bending. The following parameters were evaluated: lumbar range of motion (ROM), average maximum displacement (defined as the mean of ten points in the peak displacement region) of the functional spinal unit and the pars interarticularis, and the maximum stress and stress distribution within the pars interarticularis, intervertebral discs, and internal fixation system.

### 2.5 Statistical analysis

Statistical analyses were conducted using SPSS Statistics 26.0 (IBM Corp., Armonk, NY). Ten sampling points were selected within the peak region of the single-specimen model to calculate the mean and standard deviation, ensuring the stability of the computational output. Continuous variables are presented as the mean ± standard deviation (x̄ ± s). One-way analysis of variance (ANOVA) was used for comparisons among multiple groups. If the ANOVA indicated significant differences and the assumption of homogeneity of variance was met (as assessed by Levene’s test), *post hoc* pairwise comparisons were performed using the least significant difference (LSD) t-test. When the assumption of homogeneity of variance was violated, Welch’s ANOVA was applied, followed by the Tamhane’s T2 test for pairwise comparisons. The significance level for all statistical tests was set at a two-tailed α = 0.05.

## 3 Results

### 3.1 Mesh convergence analysis results

Convergence was evaluated based on peak von Mises stress in the fixation screw under five loading conditions. As mesh density increased, screw stress progressively converged, with differences between successive meshes falling below the 5% criterion (Ayturk and Puttlitz, 2011). This trend held true for all tissue components (cortical bone, cancellous bone, posterior elements, endplate, and disc). To balance accuracy and computational efficiency, a global mesh size of 2.0 mm combined with 1.0 mm local refinement at the screw-bone interface was selected, yielding 327,375 elements and 508,655 nodes. This configuration ensures mesh-independent results for all outcome metrics, including the peak screw stress central to our conclusions.

### 3.2 FE modeling and validation

**FIGURE 2:**
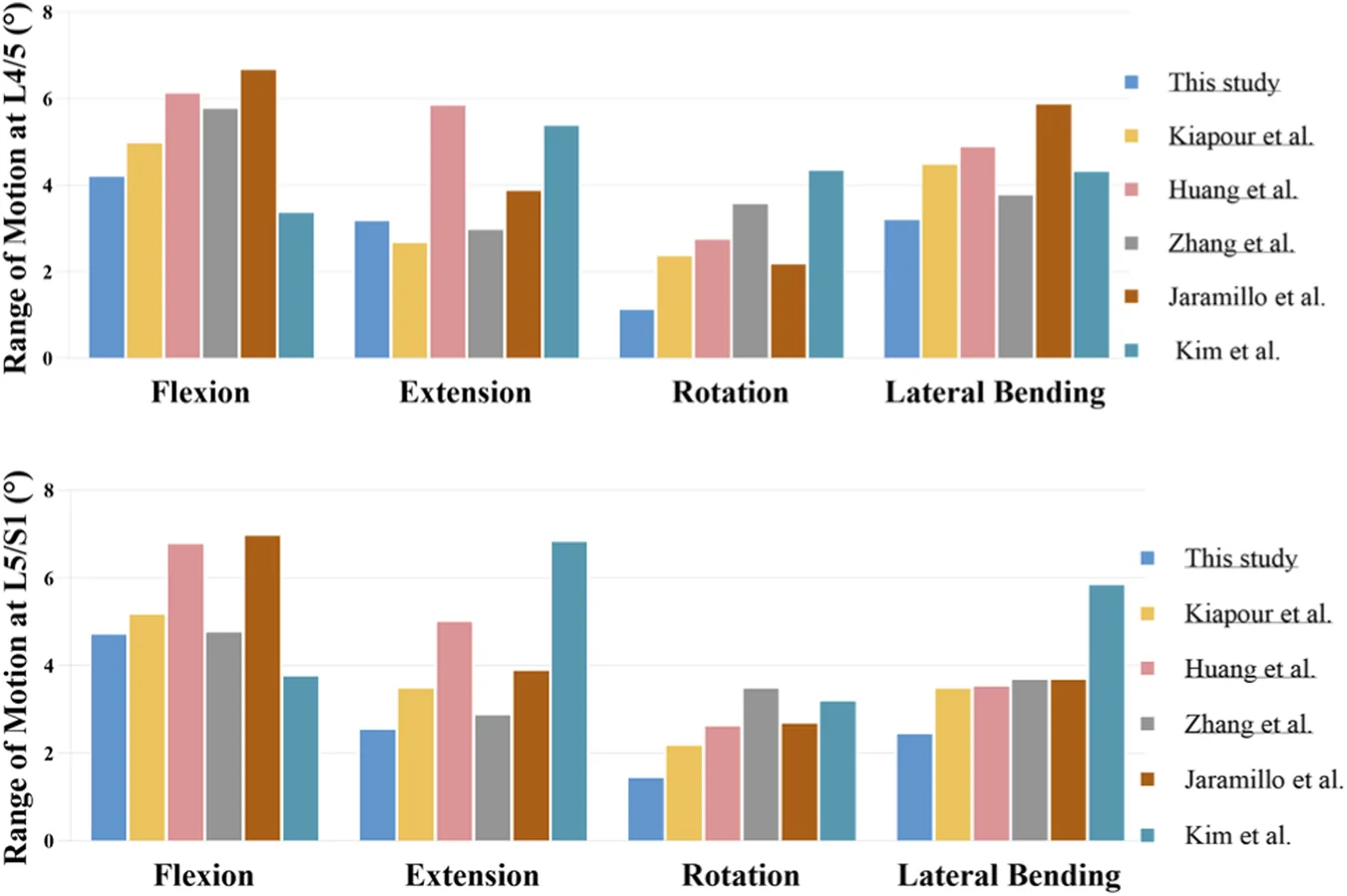
Comparison of the range of motion (ROM) of the intact model with previously reported data.

A 3D FE model of the intact L4-S1 segment (Model A) was established with a total of 327,375 elements and 508,655 nodes. Under loading conditions of 400 N axial compression and a 10 N m torsional moment, the ROMs were evaluated in four movement modes: flexion, extension, rotation, and lateral bending. At the L4/5 level, ROM measured 4.23° in flexion, 3.21° in extension, 1.15° in left rotation, and 3.23° in left lateral bending. Corresponding values at the L5/S1 level were 4.75°, 2.57°, 1.46°, and 2.46°, respectively. The segmental ROM values obtained from Model A were in close agreement with both previously reported finite element studies and *in vitro* cadaveric data (Kiapour et al., 2012; Huang et al., 2016; Zhang et al., 2021; Jaramillo et al., 2016; Kim et al., 2009) (Figure 2), thereby confirming the validity of the present FE model for subsequent analyses.

### 3.3 Overall and segment ROM

**FIGURE 3:**
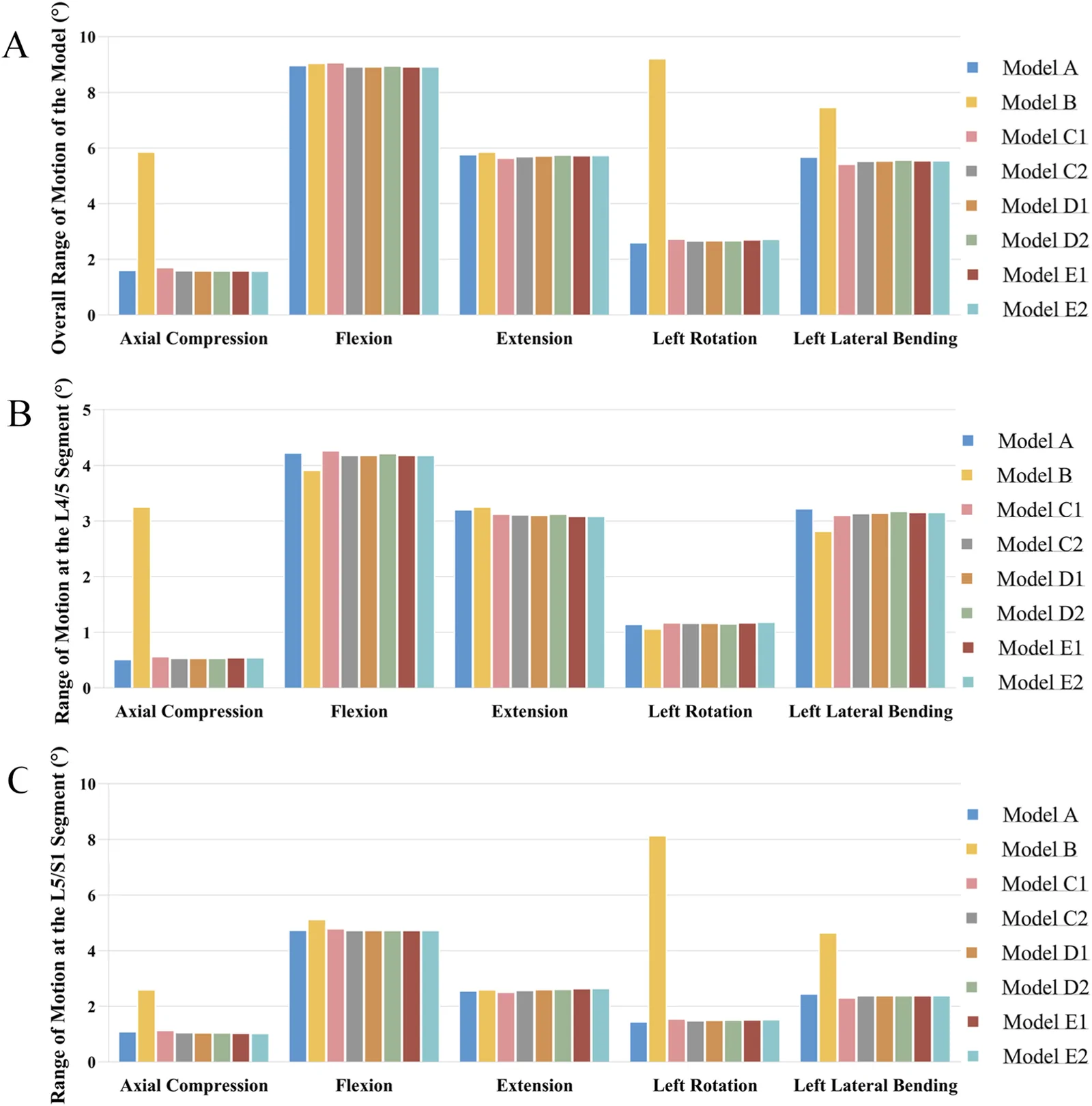
Overall range of motion **(A)**, range of motion at the L4/5 segment **(B)**, and range of motion at the L5/S1 segment **(C)** of finite element models under five loading conditions.

As shown in Figure 3A, under axial compression, left rotation, and left lateral bending, Model B showed ROM values of 5.87°, 9.22°, and 7.49°, respectively, representing increases of 262%, 255%, and 32% compared with Model A (1.62°, 2.60°, and 5.69°; all P < 0.01), indicating lumbar instability resulting from the pars defect. In flexion and extension, however, Model B’s ROM (9.06° and 5.87°) differed only slightly from Model A (8.97° and 5.78°), with increases of only 1.0% and 1.6%, likely due to greater sagittal-plane constraint from discs and ligaments. All fixation models (C1–E2) restored overall ROM to levels similar to Model A across all loading conditions, demonstrating that translaminar lag screws of different diameters (4.5 mm, 4.0 mm, 3.5 mm) and designs (solid/cannulated) can effectively reestablish overall lumbar stability following pars repair.

As shown in Figure 3B, under axial compression, the ROM at the L4/5 segment in Model B increased to 3.26°, representing a 527% rise compared with Model A (0.52°; P < 0.001). In contrast, the ROM in flexion, left rotation, and left lateral bending decreased by 7.3%, 7.0%, and 12.7%, respectively (all P < 0.05), while no significant change was observed in extension. In all fixation Models (C1–E2), the L4/5 segment ROM was restored to 0.54°–0.57°, showing no significant difference from Model A (P = 0.213). Screw diameter (4.5 mm, 4.0 mm, 3.5 mm) and cannulated design had limited influence on the L4/5 segment ROM regulation. Changes in the L5/S1 segment ROM mirrored the overall pattern, with the most pronounced alterations observed during rotation and lateral bending (Figure 3C).

### 3.4 Maximum displacement of global model

**FIGURE 4:**
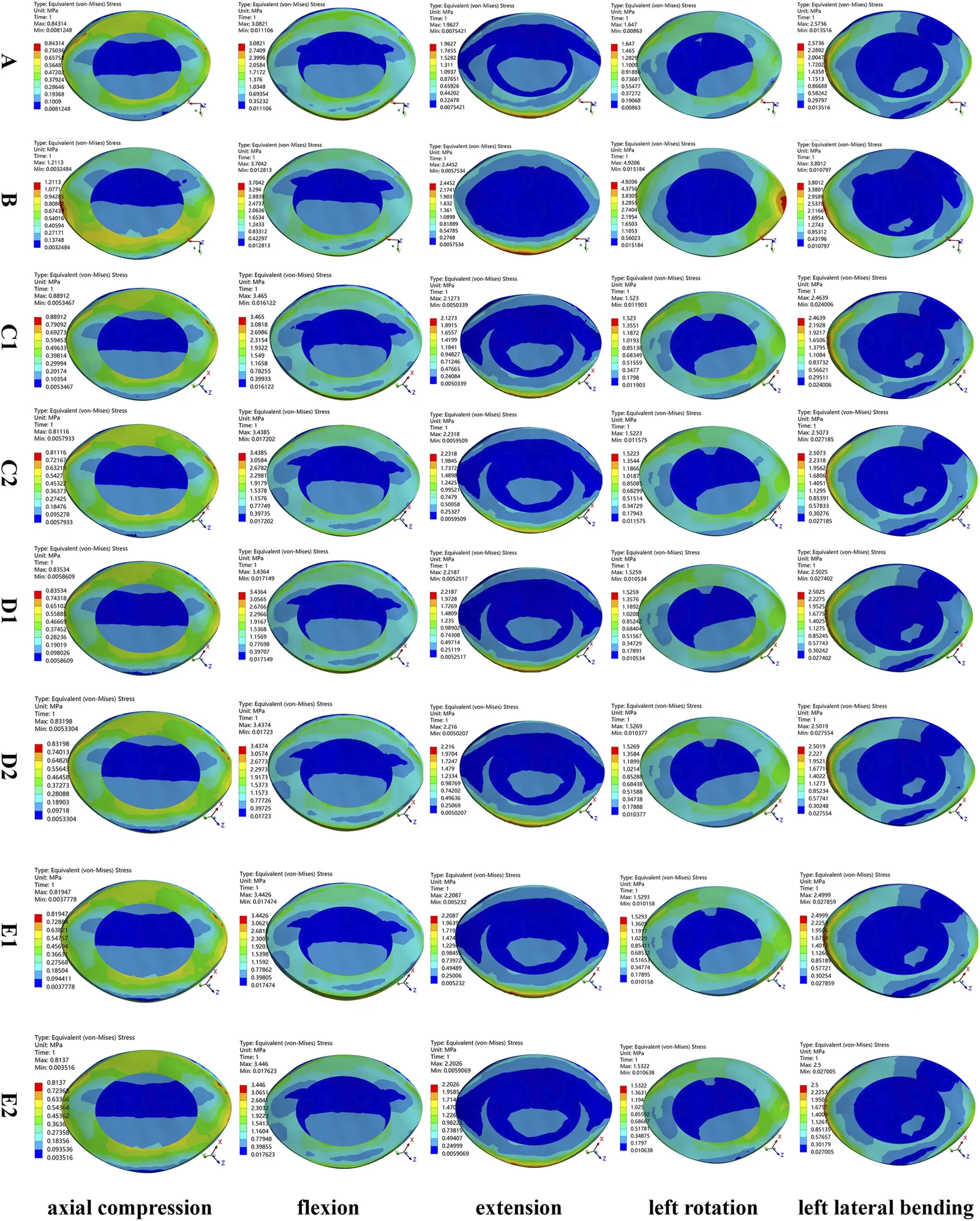
Displacement nephograms of the models under five loading conditions.

Figure 4 presents the displacement nephograms for models under five loading conditions, with red and blue areas representing maximum and minimum displacement, respectively. Under flexion and extension, the displacement nephograms showed a symmetrical pattern, indicating overall anterior or posterior displacement, whereas left rotation and lateral bending produced torsional and oblique patterns.

**FIGURE 5:**
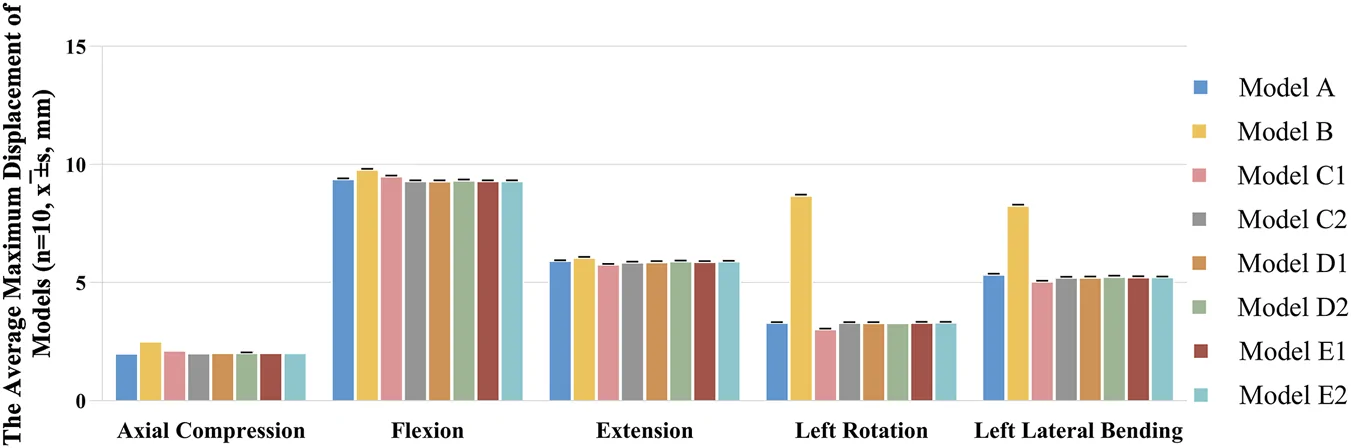
Maximum displacements of the models under five loading conditions.

The quantitative average maximum displacements are shown in Figure 5. Model B showed significantly higher displacement than Model A in all loading conditions (all P < 0.05). All fixation models (C1-E2) demonstrated significantly lower displacements than Model B in all loading conditions (all P < 0.05), restoring stability to near-intact levels. The 3.5 mm screws (E1/E2) exhibited marginally greater displacements in extension, left rotation, and left lateral bending than the larger-diameter models (C1/C2, D1/D2) (all P < 0.05). For screws of the same diameter, cannulated screws (C2/D2/E2) showed slightly greater displacements (<0.5%) in extension, left rotation, and left lateral bending than their solid counterparts (C1/D1/E1) (P < 0.05). The results of maximum displacement of models are detailed in Supplementary Table S1.

### 3.5 Maximum displacement of cancellous bone graft

**FIGURE 6:**
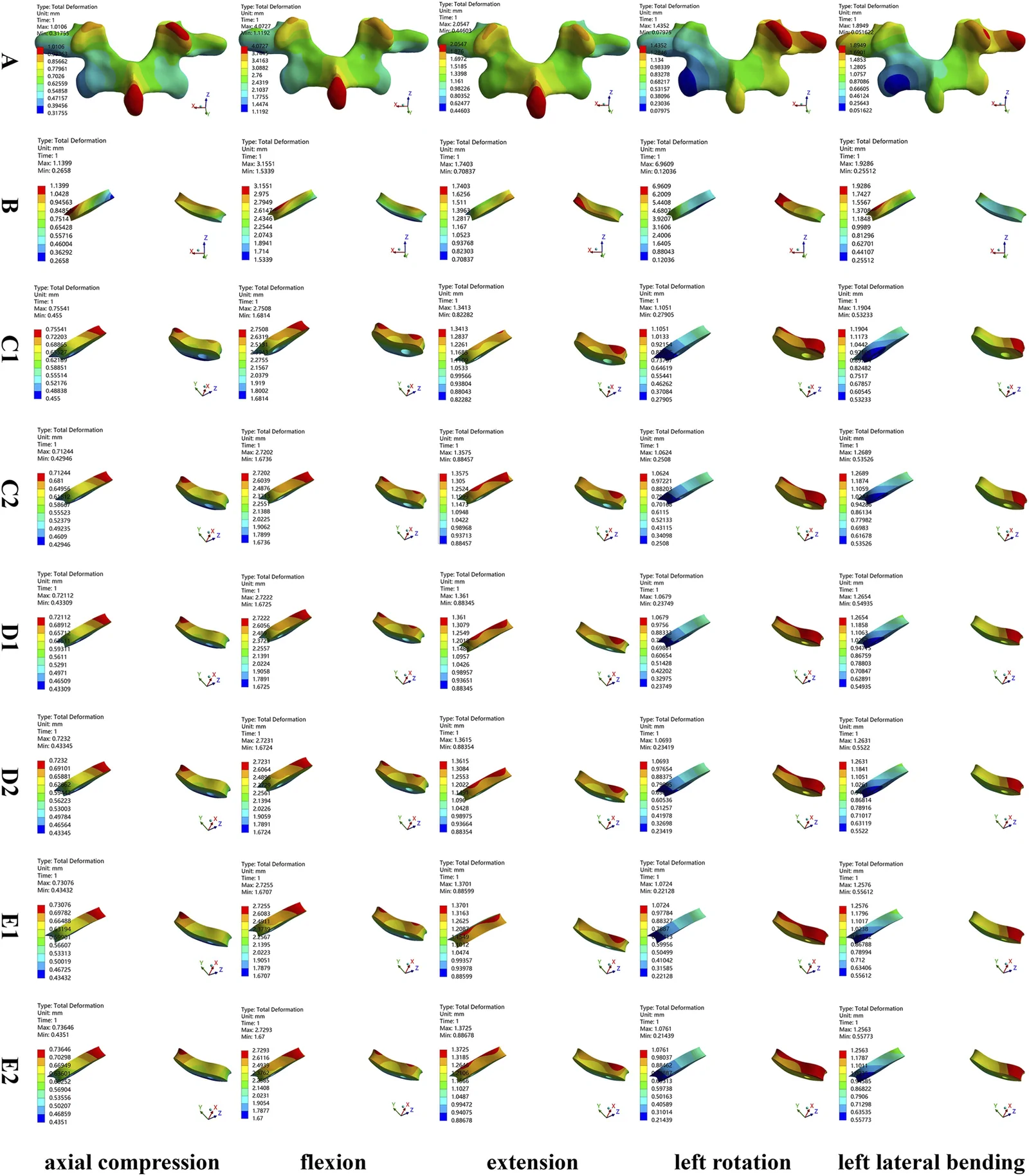
Displacement nephograms of the cancellous bone graft at the pars interarticularis under five loading conditions.

Figure 6 presents the displacement nephograms for cancellous bone graft at the pars interarticularis across all loading conditions, indicating that the location of maximum displacement was consistently at the posterosuperior aspect of the pars interarticularis.

**FIGURE 7:**
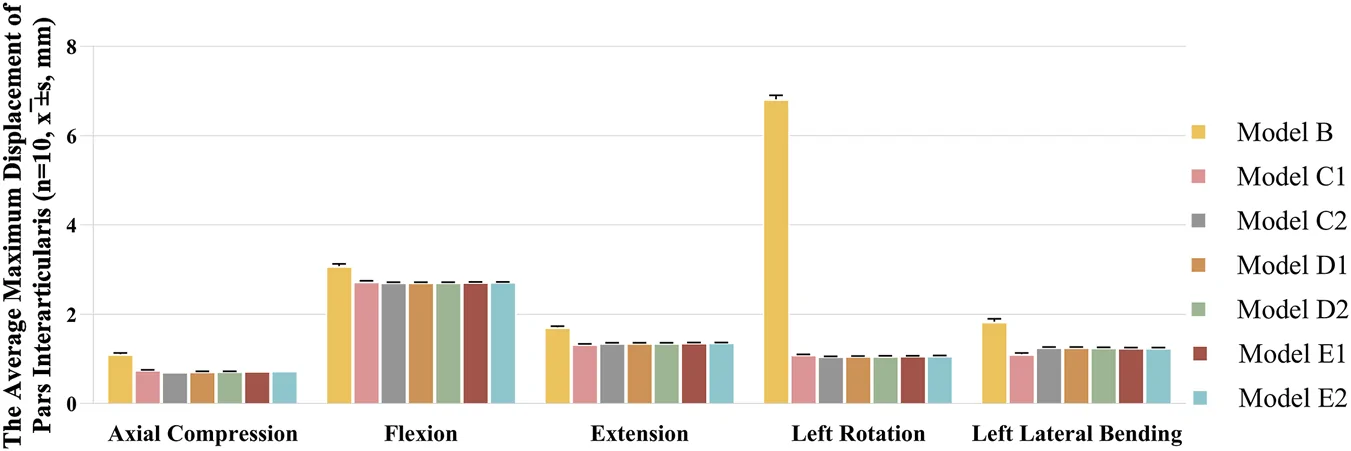
Maximum displacements of the cancellous bone graft at the pars interarticularis under five loading conditions.

As shown in Figure 7, all fixation models (C1-E2) exhibited significantly lower maximum displacement than Model B in all loading conditions (all P < 0.05). The 3.5 mm screws exhibited greater displacements in axial compression, extension, and left rotation than the larger-diameter models. For a given diameter, the cannulated design and the solid design show no significant difference in their ability to restrict the displacement of cancellous bone within the pars interarticularis. The results of maximum displacement within the pars interarticularis are detailed in Supplementary Table S2.

### 3.6 Maximum von Mises stress of the intervertebral disc

**FIGURE 8:**
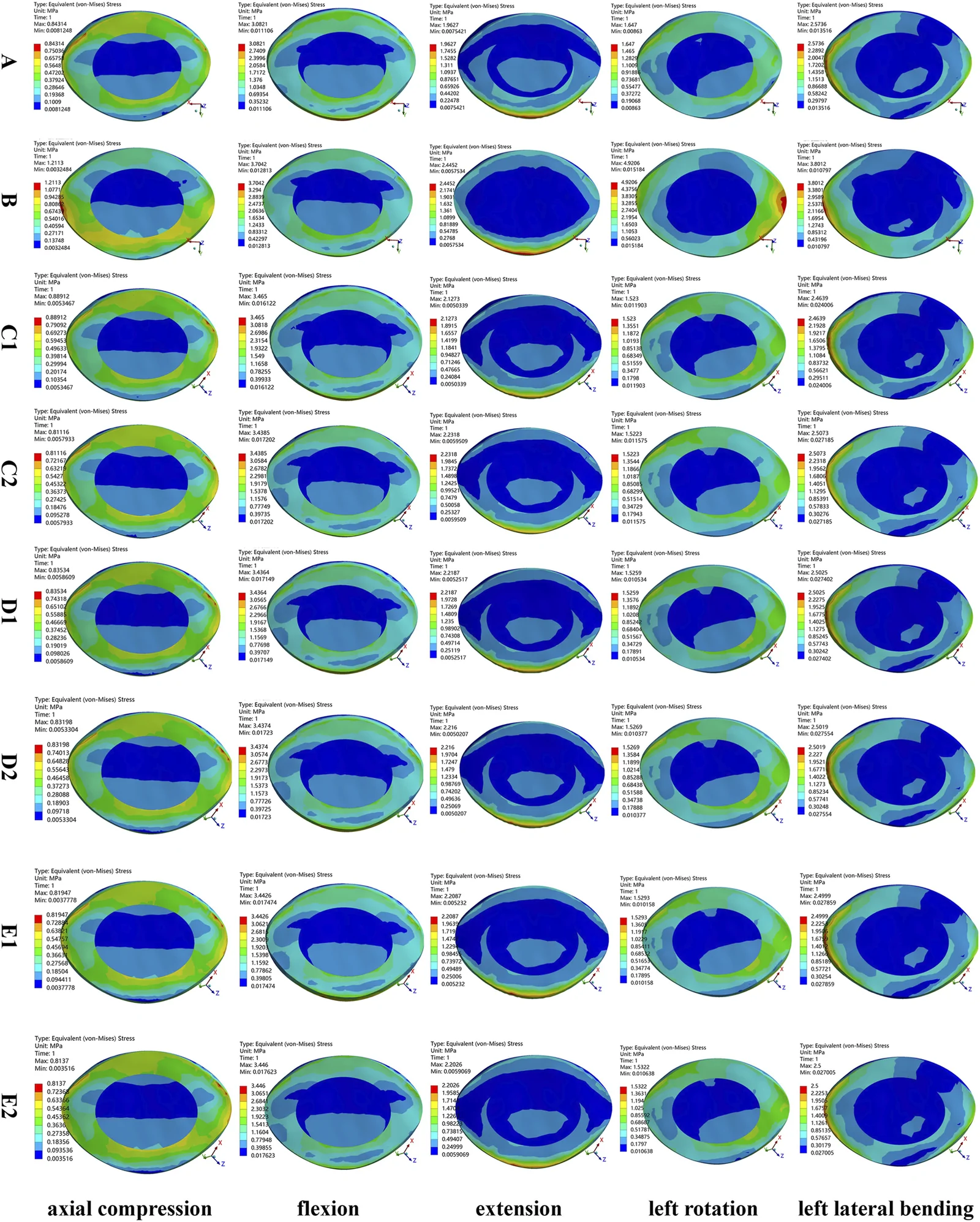
Stress nephograms of the L5/S1 intervertebral disc under five loading conditions.

Figure 8 presents the stress nephograms for the L5/S1 intervertebral disc (IVD), revealing that IVD stress distribution varied with loading mode. Under axial compression, stress concentrated peripherally; under flexion, stress concentrated on the anterior annulus; under extension, on the posterior annulus. During left rotation, stress was distributed along the right lateral and anteroposterior margins, whereas under left lateral bending, it concentrated on the left lateral margin.

**FIGURE 9:**
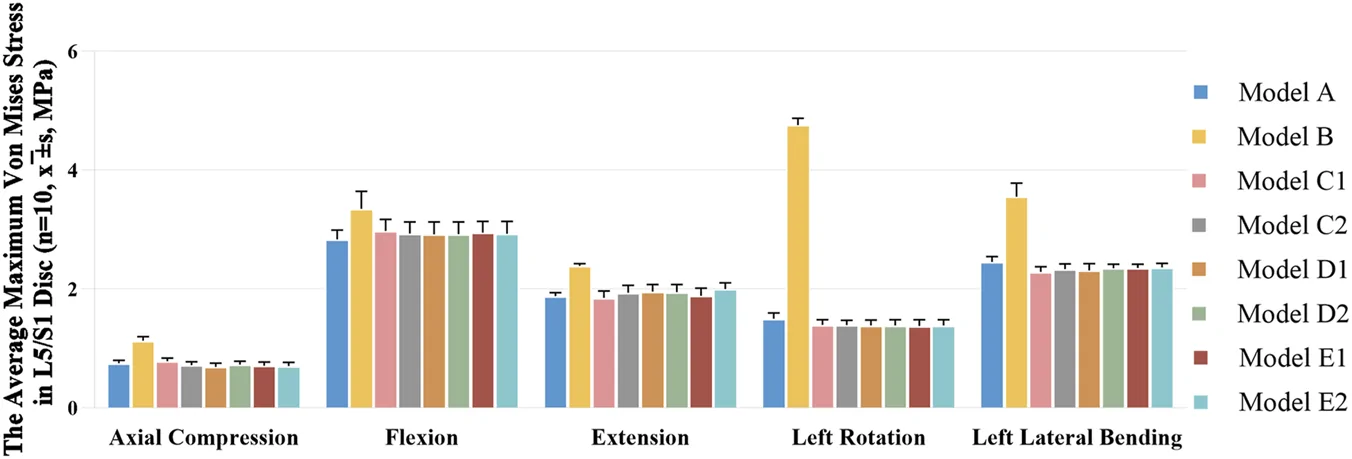
Maximum von Mises stress of the L5/S1 intervertebral disc under five loading conditions.

As shown in Figure 9, Model B exhibited significantly higher stress than the intact Model A in all motion directions, with a 216.7% increase during left rotation, indicating abnormal stress concentration. All fixation models (C1-E2) significantly reduced stress at the L5/S1. These trends are consistent with previously published findings on direct repair using the Buck technique (4.0 mm cannulated screws), in which L5–S1 disc stresses increased markedly after creation of a pars defect and were subsequently reduced to near-intact levels (annulus 125%, nucleus 120% of intact) following screw fixation, particularly under rotational loading (Sairyo et al., 2006). Reducing screw diameter (e.g., to 3.5 mm) did not significantly increase IVD stress. Notably, the 3.5 mm solid screw exhibited lower stress under extension. Between cannulated and solid screws of the same diameter, no consistent stress pattern emerged. The results of maximum von Mises stress within the intervertebral disc are detailed in Supplementary Table S3.

### 3.7 Maximum stress and stress distribution of cancellous bone graft

**FIGURE 10:**
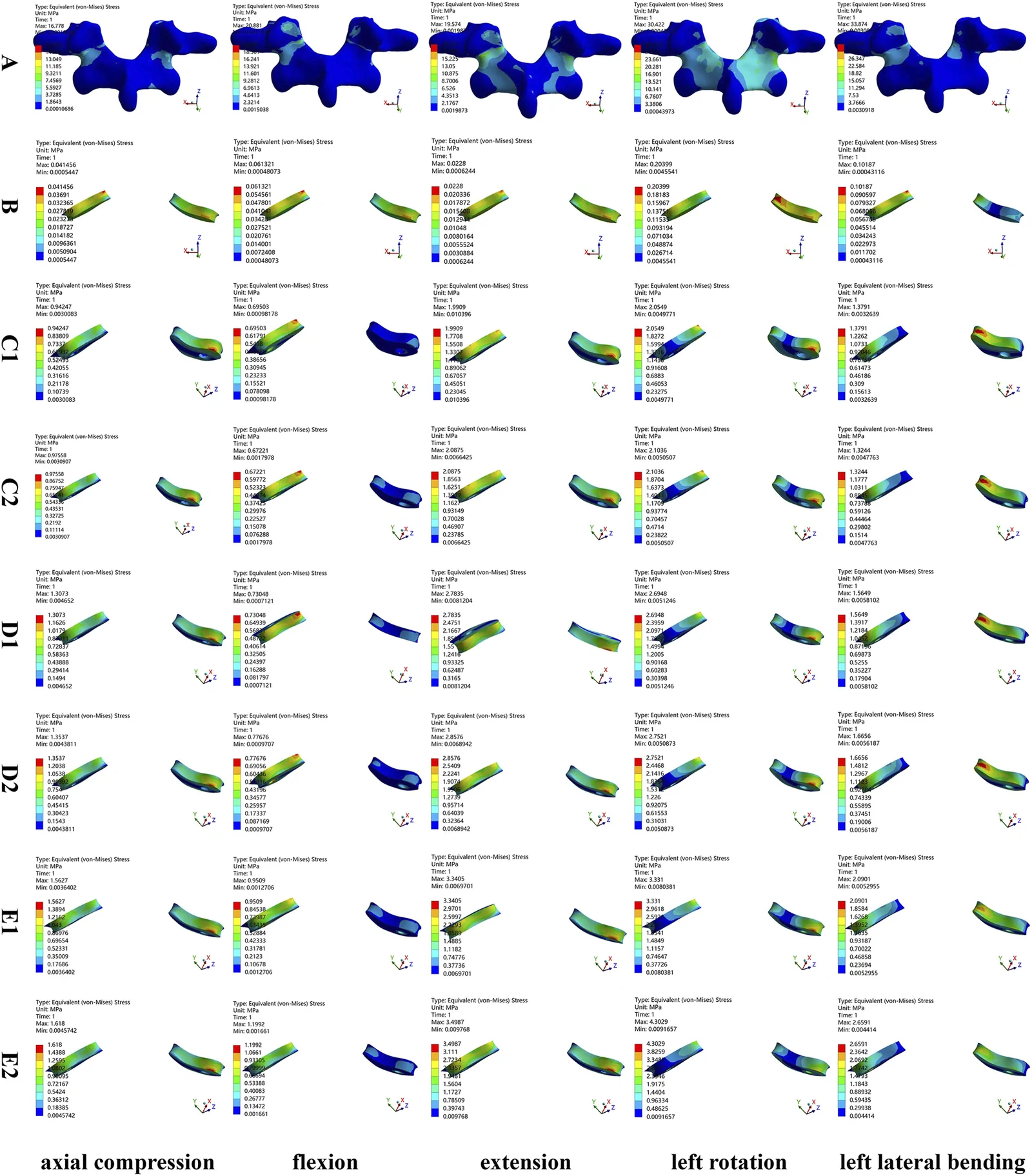
Stress nephograms of the cancellous bone graft at the pars interarticularis under five loading conditions.

Stress nephograms demonstrated that screw fixation increased the stress of cancellous bone graft within the pars interarticularis across all loading conditions, with maximum stress concentrated in the posterior region of the pars (Figure 10).

**FIGURE 11:**
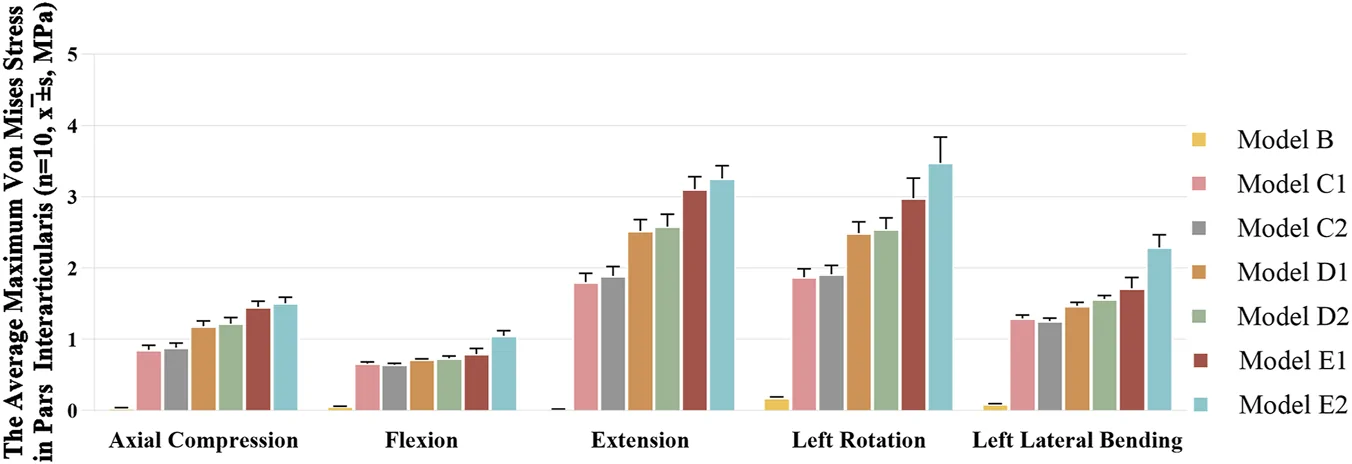
Maximum von Mises stress of the cancellous bone graft at the pars interarticularis under five loading conditions.

As shown in Figure 11, stress of cancellous bone graft within the pars interarticularis in all fixation models (C1–E2) was significantly higher than in Model B under all motions (all P < 0.05). A reduction in screw diameter significantly increased cancellous bone stress. The 3.5 mm screws (E1/E2) demonstrated significantly higher stress than all larger-diameter models in every direction (all P < 0.05). For a given diameter, cannulated screws (C2/D2/E2) exhibited slightly higher stress than their solid counterparts (C1/D1/E1) under axial compression, extension, and left rotation (all P < 0.05). The results of maximum von Mises stress within the pars interarticularis are detailed in Supplementary Table S4.

### 3.8 Maximum stress and stress distribution of internal fixation systems

**FIGURE 12:**
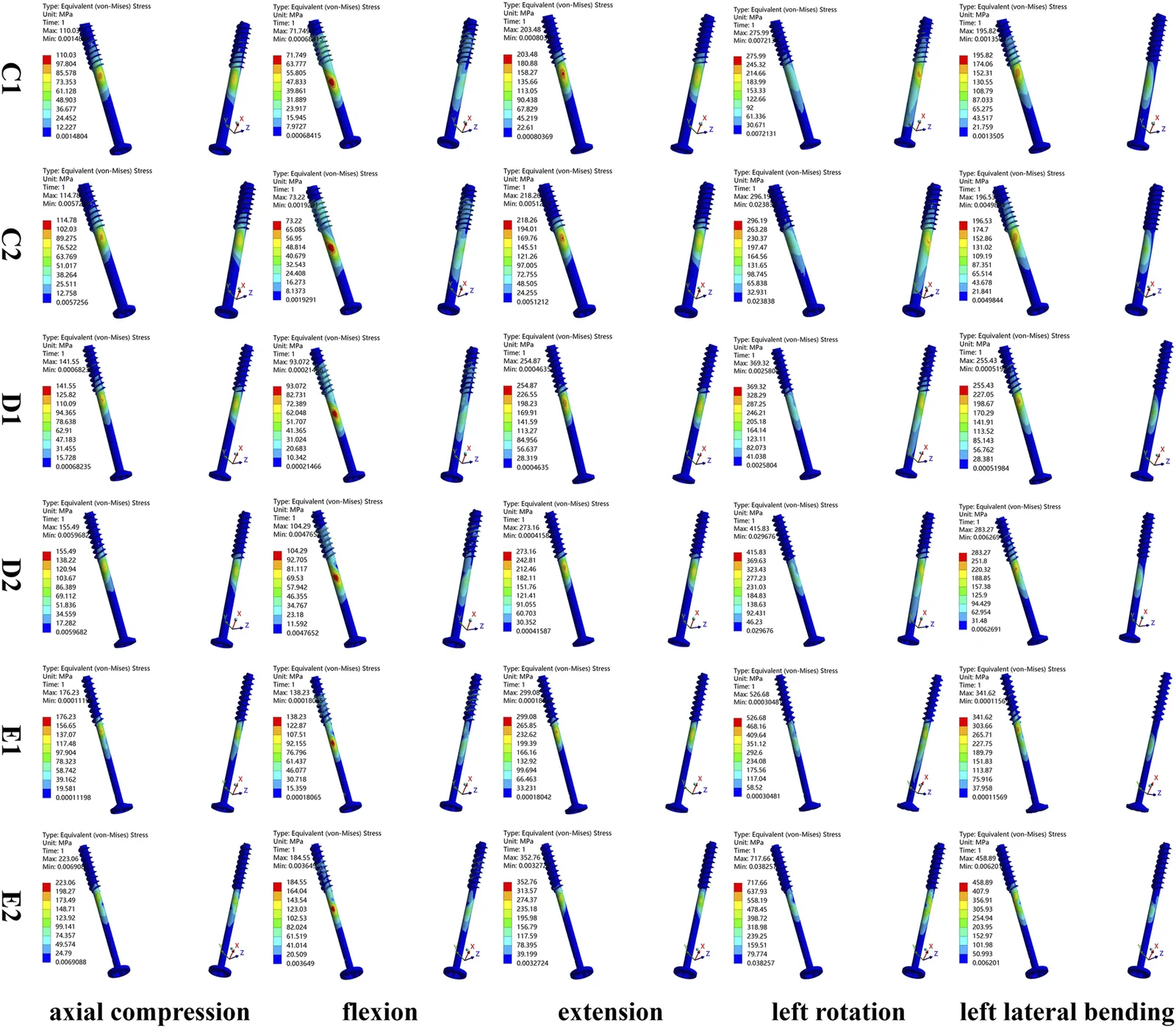
Stress nephograms of the internal fixation systems under five loading conditions.

Figure 12 presents the stress nephograms of internal fixation systems, revealing that stress was predominantly concentrated at the screw-isthmus interface.

**FIGURE 13:**
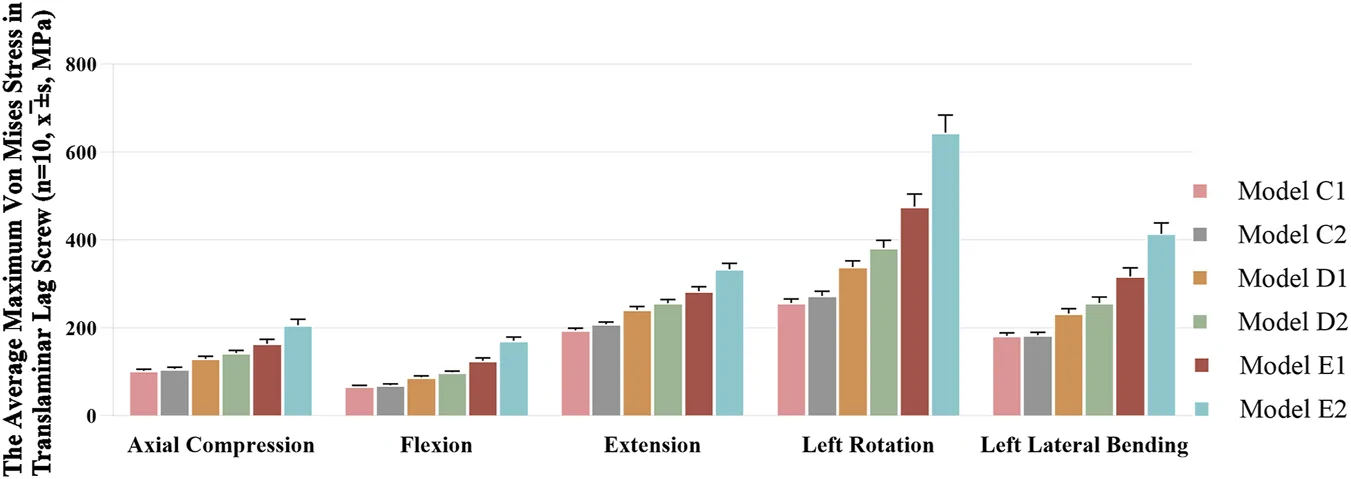
Maximum von Mises stress of the internal fixation systems under five loading conditions.

As shown in Figure 13, a reduction in screw diameter significantly increased the stress on internal fixators. During left rotation, stress on the 3.5 mm cannulated screw (E2, 644.39 MPa) was 151% higher than on the 4.5 mm solid screw (C1, 256.90 MPa) (P < 0.05). The results indicate a significant negative correlation between screw diameter and the stress on the internal fixator. For a given diameter, cannulated screws generally sustained higher stress than their solid counterparts. The 3.5 mm cannulated screw (E2) exhibited the highest stress in all motion directions, peaking at 644.39 MPa during left rotation—a 151% increase over the 4.5 mm solid screw (C1). This stress level approached the yield strength of titanium alloy (approximately 800–1,000 MPa), indicating a potential risk of fatigue fracture for small-diameter cannulated screws. The results of maximum von Mises stress within the fixation screws are detailed in Supplementary Table S5.

## 4 Discussion

This study investigated the biomechanical properties of translaminar lag screws with different diameters (4.5, 4.0, 3.5 mm) and structural designs (solid/cannulated) for the repair of L5 bilateral spondylolysis. Using finite element analysis, the ROM (overall and segmental) was compared among all models under five loading conditions. Additionally, the maximum displacement of the global model and cancellous bone graft in the pars interarticularis, as well as the maximum stress in the intervertebral disc, cancellous bone graft, and the internal fixation system, were evaluated. The displacement and stress distribution characteristics were further compared via displacement and stress nephograms, respectively.

The spondylolysis model (B) exhibited significant biomechanical abnormalities compared to the intact model (A), manifesting as an abnormal ROM, increased displacement, and disordered stress distribution in the intervertebral disc. All internal fixation models (C1-E2) effectively normalized vertebral ROM, restricted excessive displacement, and improved the stress distribution in the intervertebral disc. The observed trends—increased disc stress after spondylolysis and its partial normalization after direct fixation—are in agreement with earlier FE studies reporting similar percentage increases in annulus and nucleus stresses at the defect and adjacent levels, which were reduced following Buck’s repair technique (Sairyo et al., 2006).

When comparing the effects of different screw designs on spinal stability, it was found that all screw designs effectively restored both the overall and segmental ROM, limiting displacement to near-normal levels. The results indicated that screw diameter and cannulated design had limited influence on the ROM. However, under multiple loading conditions, larger-diameter and solid designs demonstrated smaller displacement, suggesting that the 4.5 mm solid screw performed best in restricting displacement, whereas the 3.5 mm screw still met fundamental biomechanical requirements.

When comparing the impact of different screw designs on the displacement and stress of cancellous bone graft in the pars interarticularis, it was found that all screw designs improved the stability of the cancellous bone graft. The results showed that under multiple loading conditions, larger-diameter screws exhibited a trend of less displacement in the cancellous bone of the pars interarticularis. Increasing screw diameter significantly reduced stress, while the cannulated design showed no significant difference in restricting cancellous bone displacement but increased cancellous bone stress compared with the solid design. These findings are consistent with the previous study reporting that increasing screw diameter reduces cancellous bone stress and displacement (Qi et al., 2011). This indicates that the 4.5 mm solid screw performed best in sharing cancellous bone stress and controlling displacement, providing an ideal mechanical environment for bone graft fusion.

When comparing the effects of different screw designs on the stress distribution of the intervertebral disc and internal fixation, it was found that the internal fixation system effectively restored near-physiological IVD stress distribution. Screw diameter and design had a relatively minor, direction-specific influence on disc stress, with the 3.5 mm solid screw performing best in extension. The stress nephograms of the internal fixation showed that stress was more concentrated in the screw segment fixing the pars. A decrease in screw diameter led to a significant increase in the stress on the implant. Among screws of the same diameter, cannulated screws generally exhibited higher stress than solid screws, which is consistent with a previous study (Chang et al., 2017). The 4.5 mm solid screw carried the lowest risk of screw breakage.

This study has several limitations. First, while the FE models effectively simulated spinal kinematics and mechanical properties, they represent a simplification of clinical reality. Key biological factors, such as the complex constraints of spinal soft tissues and the dynamic influence of surrounding musculature, were not incorporated. Second, the investigation was confined to a single-level lumbar spondylolysis model. Future studies should incorporate multi-level pathological models to enable a more comprehensive assessment of the biomechanical behavior of translaminar lag screw fixation. Third, although multiple implant configurations were compared, the study did not encompass the full spectrum of potential clinical scenarios. Finally, the FE model was reconstructed from the CT data of a single patient. Given the considerable anatomical variability in the lumbar spine, our quantitative results may not be directly generalizable to the entire population.

## 5 Conclusion

All screw designs restored spinal stability to near-intact levels in the present FE model. While variations in screw diameter and structural design minimally affected overall stability, they significantly influenced implant stress. The 4.5 mm solid screw demonstrated the lowest stress levels and effective control of graft displacement, suggesting a favorable biomechanical environment for achieving fusion within this specific anatomical context. Conversely, while the cannulated screw increased implant stress, it did not adversely affect disc mechanics or graft stability, indicating its potential utility in minimally invasive scenarios where anatomical constraints limit screw size. However, given the inherent limitations of a single-patient model, these findings should be interpreted as preliminary biomechanical evidence rather than definitive clinical guidelines. Future multi-specimen studies and clinical trials are required to validate these results and establish quantitative criteria for individualized implant selection.

## Data Availability

The original contributions presented in the study are included in the article/Supplementary Material, further inquiries can be directed to the corresponding author.
