# Supplementary material for: Three-dimensional finite element analysis of translaminar lag screw designs for L5 bilateral spondylolysis
Source: Front Bioeng Biotechnol. 2026 Aug 13;14:1882221. doi: 10.3389/fbioe.2026.1882221 (PMC13519390; doi:10.3389/fbioe.2026.1882221)
Supplement: Supplementary file 1 [file Table1.docx]

Supplementary table 1. Maximum displacement of global model (n=10, x ̅±s, mm)

| Model | Axial compression* | Flexion^#^ | Extension^#^ | Left rotation^#^ | Left lateral bending* |
| --- | --- | --- | --- | --- | --- |
| A | 2.018±0.001 | 9.405±0.012 | 5.937±0.009 | 3.319±0.003 | 5.356±0.014 |
| B | 2.527±0.002^a^ | 9.801±0.021^a^ | 6.073±0.009^a^ | 8.707±0.025^a^ | 8.279±0.017^a^ |
| C1 | 2.149±0.002^ab^ | 9.524±0.005^ab^ | 5.783±0.004^ab^ | 3.046±0.004^ab^ | 5.066±0.007^ab^ |
| C2 | 2.033±0.002^abc^ | 9.314±0.013^abc^ | 5.865±0.012^abc^ | 3.318±0.006^bc^ | 5.228±0.016^abc^ |
| D1 | 2.039±0.001^abcd^ | 9.309±0.021^abc^ | 5.879±0.022^abc^ | 3.314±0.007^bc^ | 5.236±0.015^abc^ |
| D2 | 2.038±0.002^abcd^ | 9.345±0.017^abcde^ | 5.920±0.013^bcde^ | 3.313±0.003^abc^ | 5.267±0.018^abcde^ |
| E1 | 2.040±0.001^abcdf^ | 9.317±0.013^abcf^ | 5.900±0.005^abcdf^ | 3.326±0.006^bcef^ | 5.241±0.020^abcf^ |
| E2 | 2.043±0.001^abcdefg^ | 9.315±0.019^abc^ | 5.913±0.006^abcdeg^ | 3.333±0.004^abcdef^ | 5.250±0.010^abcdf^ |
| *F* value | 148500.401**^*^** | 1113.225**^#^** | 1461.278**^#^** | 56996.584**^#^** | 50377.072* |
| *P* value | ＜0.001 | ＜0.001 | ＜0.001 | ＜0.001 | ＜0.001 |

Supplementary table 2. Maximum displacement of cancellous bone graft (n=10, x ̅±s, mm)

| Model | Axial compression^#^ | Flexion^#^ | Extension^#^ | Left rotation^#^ | Left lateral bending^#^ |
| --- | --- | --- | --- | --- | --- |
| B | 1.112±0.020 | 3.078±0.055 | 1.712±0.020 | 6.815±0.087 | 1.836±0.063 |
| C1 | 0.753±0.002^b^ | 2.734±0.017^b^ | 1.333±0.006^b^ | 1.098±0.005^b^ | 1.108±0.029^b^ |
| C2 | 0.711±0.001^bc^ | 2.714±0.006^b^ | 1.353±0.006^bc^ | 1.056±0.004^bc^ | 1.260±0.007^bc^ |
| D1 | 0.718±0.004^bcd^ | 2.717±0.004^b^ | 1.354±0.006^bc^ | 1.063±0.004^bcd^ | 1.259±0.005^bc^ |
| D2 | 0.721±0.001^bcd^ | 2.717±0.006^b^ | 1.355±0.004^bc^ | 1.065±0.004^bcd^ | 1.257±0.005^bc^ |
| E1 | 0.729±0.001^bcdef^ | 2.720±0.004^b^ | 1.364±0.005^bcdef^ | 1.069±0.003^bcde^ | 1.251±0.006^bc^ |
| E2 | 0.736±0.002^bcdefg^ | 2.724±0.005^bde^ | 1.366±0.004^bcdef^ | 1.073±0.002^bcdef^ | 1.251±0.004^bcde^ |
| *F* value | 985.017**^#^** | 67.799**^#^** | 517.887**^#^** | 6497.677**^#^** | 169.437^#^ |
| *P* value | ＜0.001 | ＜0.001 | ＜0.001 | ＜0.001 | ＜0.001 |

Supplementary table 3. Maximum Von Mises stress of L5/S1 intervertebral disc (n=10, x ̅±s, MPa)

| Model | Axial compression* | Flexion* | Extension* | Left rotation* | Left lateral bending^#^ |
| --- | --- | --- | --- | --- | --- |
| A | 0.751±0.047 | 2.834±0.155 | 1.877±0.060 | 1.501±0.094 | 2.453±0.088 |
| B | 1.129±0.070^a^ | 3.349±0.294^a^ | 2.384±0.038^a^ | 4.754±0.115^a^ | 3.553±0.225^a^ |
| C1 | 0.783±0.055^b^ | 2.974±0.192^b^ | 1.849±0.114^b^ | 1.396±0.083^ab^ | 2.288±0.085^ab^ |
| C2 | 0.718±0.059^bc^ | 2.930±0.197^b^ | 1.935±0.125^b^ | 1.390±0.082^ab^ | 2.335±0.086^b^ |
| D1 | 0.690±0.063^abc^ | 2.916±0.208^b^ | 1.953±0.120^bc^ | 1.385±0.092^ab^ | 2.310±0.114^b^ |
| D2 | 0.729±0.055^bc^ | 2.922±0.205^b^ | 1.944±0.130^b^ | 1.384±0.095^ab^ | 2.349±0.067^b^ |
| E1 | 0.707±0.065^bc^ | 2.946±0.190^b^ | 1.885±0.127^b^ | 1.379±0.102^ab^ | 2.352±0.064^b^ |
| E2 | 0.705±0.061^bc^ | 2.928±0.207^b^ | 1.999±0.101^abcg^ | 1.388±0.092^ab^ | 2.361±0.067^b^ |
| F value | 59.161* | 5.583* | 25.480* | 1563.606* | 37.972# |
| P value | ＜0.001 | ＜0.001 | ＜0.001 | ＜0.001 | ＜0.001 |

Supplementary table 4.	Maximum stress of cancellous bone graft (n=10, x ̅±s, MPa)

| Model | Axial compression^#^ | Flexion^#^ | Extension^#^ | Left rotation^#^ | Left lateral bending^#^ |
| --- | --- | --- | --- | --- | --- |
| B | 0.038±0.002 | 0.058±0.002 | 0.020±0.002 | 0.181±0.012 | 0.093±0.005 |
| C1 | 0.853±0.060^b^ | 0.659±0.021^b^ | 1.802±0.122^b^ | 1.871±0.118^b^ | 1.293±0.049^b^ |
| C2 | 0.883±0.062^b^ | 0.643±0.018^b^ | 1.890±0.130^b^ | 1.914±0.122^b^ | 1.256±0.042^b^ |
| D1 | 1.183±0.074^bcd^ | 0.712±0.012^bcd^ | 2.522±0.157^bcd^ | 2.492±0.155^bcd^ | 1.470±0.048^bcd^ |
| D2 | 1.220±0.085^bcd^ | 0.733±0.031^bcd^ | 2.582±0.171^bcd^ | 2.546±0.157^bcd^ | 1.566±0.049^bcde^ |
| E1 | 1.455±0.081^bcdef^ | 0.790±0.082^bcd^ | 3.108±0.171^bcdef^ | 2.981±0.280^bcdef^ | 1.715±0.153^bcde^ |
| E2 | 1.508±0.083^bcdef^ | 1.048±0.070^bcdefg^ | 3.255±0.179^bcdef^ | 3.475±0.361^bcdef^ | 2.288±0.179^bcdefg^ |
| *F* value | 2083.472**^#^** | 7477.381**^#^** | 2274.016**^#^** | 1468.031**^#^** | 4680.658^#^ |
| *P* value | ＜0.001 | ＜0.001 | ＜0.001 | ＜0.001 | ＜0.001 |

Supplementary table 5.	Maximum stress of internal ﬁxation systems (n=10, x ̅±s, MPa)

| Model | Axial compression^#^ | Flexion^#^ | Extension^#^ | Left rotation^#^ | Left lateral bending^#^ |
| --- | --- | --- | --- | --- | --- |
| C1 | 102.36±3.54 | 67.02±2.44 | 194.27±5.01 | 256.90±8.77 | 182.08±6.43 |
| C2 | 106.55±4.03 | 69.17±2.96 | 207.90±5.59^c^ | 272.69±10.83^c^ | 183.28±6.61 |
| D1 | 129.75±5.65^cd^ | 87.78±2.86^cd^ | 241.11±7.38^cd^ | 338.09±14.40^cd^ | 232.72±10.81^cd^ |
| D2 | 142.98±5.69^cde^ | 98.77±2.50^cde^ | 256.48±8.02^cde^ | 381.84±17.25^cde^ | 256.87±13.17^cde^ |
| E1 | 164.28±10.08^cdef^ | 125.48±6.13^cdef^ | 283.23±10.25^cdef^ | 476.10±28.52^cdef^ | 316.72±19.61^cdef^ |
| E2 | 206.45±13.10^cdefg^ | 170.77±8.30^cdefg^ | 333.14±13.21^cdefg^ | 644.39±39.96^cdefg^ | 414.29±24.27^cdefg^ |
| *F* value | 212.488**^#^** | 478.015**^#^** | 300.119**^#^** | 310.463**^#^** | 274.512^#^ |
| *P* value | ＜0.001 | ＜0.001 | ＜0.001 | ＜0.001 | ＜0.001 |

Note: Superscript letters denote pairwise comparisons: a, compared with Model A (P< 0.05); b, compared with Model B (P< 0.05); c, compared with C1 (P< 0.05); d, compared with C2 (P< 0.05); e, compared with D1 (P< 0.05); f, compared with D2 (P< 0.05); g, compared with E1 (P< 0.05). Statistical analyses were performed as follows: when homogeneity of variance was confirmed (*), the Least Significant Difference (LSD) test was used; when homogeneity of variance was violated (#), the Welch ANOVA was applied, followed by the Tamhane's T2 post hoc test.
